# Adjunctive clindamycin for cellulitis: a clinical trial comparing flucloxacillin with or without clindamycin for the treatment of limb cellulitis

**DOI:** 10.1136/bmjopen-2016-013260

**Published:** 2017-03-17

**Authors:** Richard Brindle, O Martin Williams, Paul Davies, Tim Harris, Heather Jarman, Alastair D Hay, Peter Featherstone

**Affiliations:** 1 Microbiology and Infectious Diseases, Bristol Royal Infirmary, Bristol, UK; 2 General Practice Support Unit, Bristol Royal Infirmary, Bristol, UK; 3 Department of Emergency Medicine, Royal London Hospital, London, UK; 4 Department of Emergency Medicine, St George's University Hospitals, London, UK; 5 Centre for Academic Primary Care, University of Bristol, Bristol, UK; 6 Acute Medicine Unit, Queen Alexandra Hospital, Portsmouth, UK

**Keywords:** cellulitis, erysipelas, flucloxacillin, clindamycin, limb, diarrhoea

## Abstract

**Objective:**

To compare flucloxacillin with clindamycin to flucloxacillin alone for the treatment of limb cellulitis.

**Design:**

Parallel, double-blinded, randomised controlled trial.

**Setting:**

Emergency department attendances and general practice referrals within 20 hospitals in England.

**Interventions:**

Flucloxacillin, at a minimum of 500 mg 4 times per day for 5 days, with clindamycin 300 mg 4 times per day for 2 days given orally versus flucloxacillin given alone.

**Main outcome measures:**

The primary outcome was improvement at day 5. This was defined as being afebrile with either a reduction in affected skin surface temperature or a reduction in the circumference of the affected area. Secondary outcomes included resolution of systemic features, resolution of inflammatory markers, recovery of renal function, reduction in the affected area, decrease in pain, return to work or normal activities and the absence of increased side effects.

**Results:**

410 patients were included in the trial. No significant difference was seen in improvement at day 5 for flucloxacillin with clindamycin (136/156, 87%) versus flucloxacillin alone (140/172, 81%)—OR 1.55 (95% CI 0.81 to 3.01), p=0.174. There was a significant difference in the number of patients with diarrhoea at day 5 in the flucloxacillin with clindamycin allocation (34/160, 22%) versus flucloxacillin alone (16/176, 9%)—OR 2.7 (95% CI 1.41 to 5.07), p=0.002. There was no clinically significant difference in any secondary outcome measures. There was no significant difference in the number of patients stating that they had returned to normal activities at the day 30 interview in the flucloxacillin with clindamycin allocation (99/121, 82%) versus flucloxacillin alone (104/129, 81%)—adjusted OR 0.90 (95% CI 0.44 to 1.84).

**Conclusions:**

The addition of a short course of clindamycin to flucloxacillin early on in limb cellulitis does not improve outcome. The addition of clindamycin doubles the likelihood of diarrhoea within the first few days.

**Trial registration number:**

NCT01876628, Results.

**Strengths and limitations of this study**

- This was a double-blind randomised multicentre study and the first to examine the effect of adjunctive clindamycin with β-lactam therapy for cellulitis.
- Patients were recruited from general practice, emergency department patients and inpatients and are thus a representative population.
- The study lost 18% of the patients by the first follow-up visit but the characteristics of these patients were similar in both drug allocations.

## Introduction

Cellulitis is a common acute skin infection occurring anywhere on the body, which causes pain, swelling and erythema. It cannot be reliably distinguished from erysipelas which may be considered a form of cellulitis.^1^ Cellulitis may be accompanied by fever and other systemic features. A UK study in 1992^2^ estimated a cellulitis incidence rate of 16.4 per 1000 person-years in patients presenting to general practice. In England alone during 2012, people admitted with a diagnosis of cellulitis took up 400 000 bed days.^3^

Microbiological studies are positive in a variable proportion of people who present to hospital with erysipelas or cellulitis. The use of latex agglutination techniques and direct immunofluorescence on skin biopsy specimens increases the yield and has shown that β haemolytic streptococci, usually group A streptococci (GAS) or group G, represent the most prominent bacteria in studies of cellulitis and erysipelas, accounting for almost 80% of isolated organisms.^4^ ^5^ Molecular testing^6^ and serology^7^ have been found to be of variable value but can improve the yield, especially if the patient has received antibiotics.^8^ It is probable that *Staphylococcus aureus* is not the causative agent in most cases where it was isolated, adopting an opportunistic bystander role. This is supported by a study which demonstrated no extra benefit when adding an antibiotic active against meticillin-resistant *S. aureus* (MRSA) in the presence of MRSA carriage.^9^

Standard therapy is with a β-lactam antibiotic, commonly flucloxacillin or a cefalosporin, which, while effective,^10^ often leaves the patient with significant skin damage which takes many weeks to heal. β-lactams need a growing or dividing bacterium in order to work. Clindamycin, a protein-inhibiting antibiotic, inhibits toxin production and is able to kill intracellular streptococci.^11^ ^12^ It is these toxins which are responsible for local damage and systemic features.^13^ Protein-inhibiting antibiotics are part of recommended therapy for invasive GAS infections such as necrotising fasciitis and pleural empyema, but there is conflicting evidence of any benefit^14^ ^15^ and there are no clinical trials to support it. The evidence for an effect in reducing toxin has been predominantly in vitro and even in vitro studies show antagonism under some conditions,^16^ but one retrospective and one prospective review showed the benefit of clindamycin in invasive infections caused by GAS.^17^ ^18^

Clindamycin is a recommended treatment for cellulitis in the British National Formulary;^19^ it is a lincosamide and is also active against some macrolide (eg, clarithromycin) resistant strains of streptococci and staphylococci. It was the second most common treatment for cellulitis in a survey of Canadian hospitals^20^ and clindamycin or clarithromycin featured in every 1 of 23 guidelines examined as part of a review of treatment of cellulitis in the south west of England.^21^ If clindamycin is an effective adjuvant in serious streptococcal infections, its benefit should be detectable in cellulitis. A reduction in toxin production should lead to a reduction in the severity of infection and result in less pain, more rapid resolution, improved short-term health-related quality of life and a more rapid return to normal activity.

This trial was designed to determine whether clindamycin has an additional beneficial effect in cellulitis. Clear evidence that clindamycin has no benefit would have implications for the use of it as adjunctive or sequential therapy in cellulitis. Failure to demonstrate benefit would also suggest that the present recommendations for its use in invasive GAS infections need to be re-evaluated.

## Methods

### Trial design and participants

We conducted a double-blind, randomised, placebo-controlled trial from October 2013 to December 2015, with 1:1 parallel group allocation. Potential participants were screened from emergency departments, hospital inpatients and referrals to hospital from general practice (family physicians) from 20 hospitals in England.

**Figure 1:**
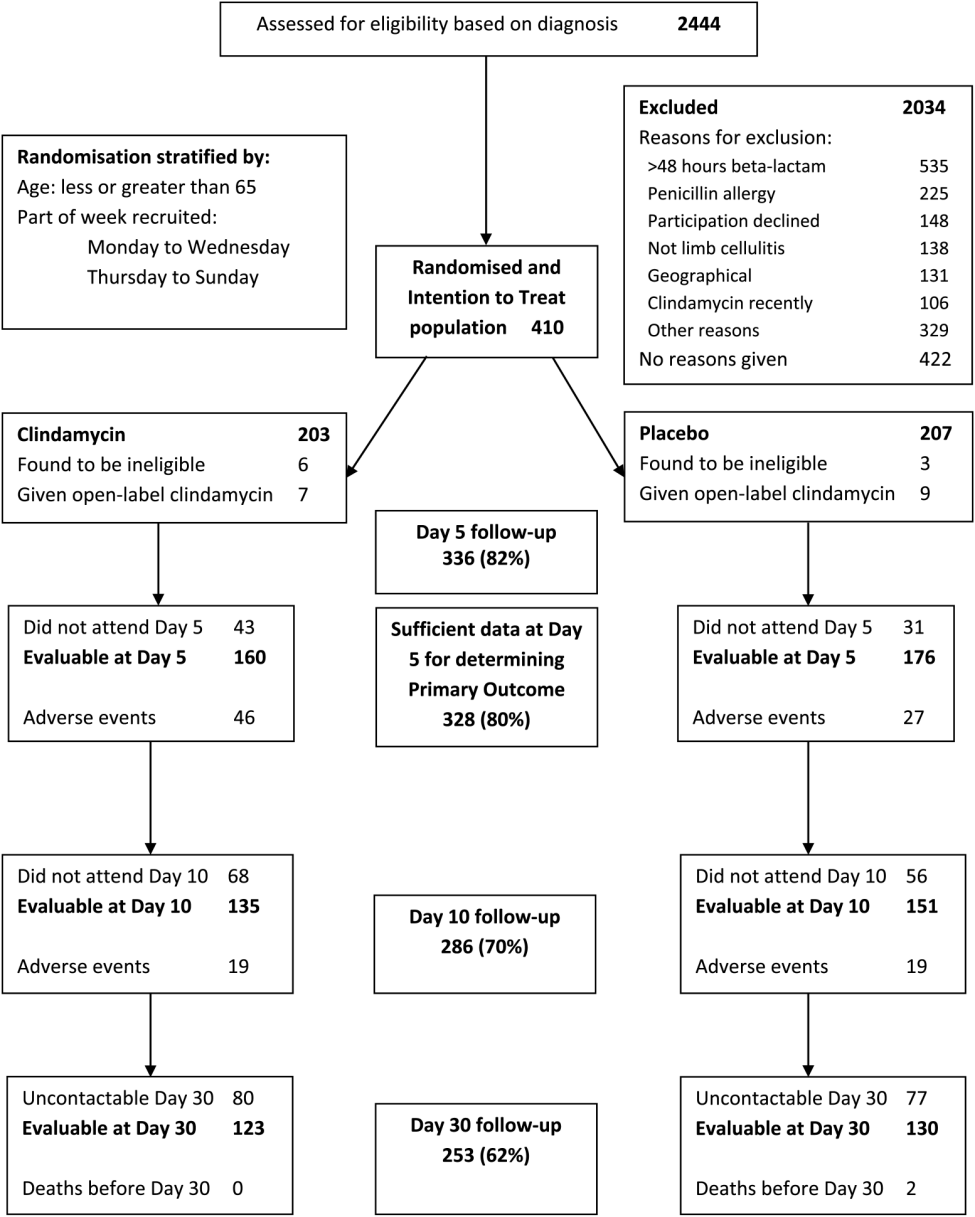
Flow chart summarising the numbers of patients screened for eligibility and numbers present at each follow-up.

The diagnosis of cellulitis was supported using a set of criteria established for the PATCH trials on the prevention of recurrent cellulitis.^22^ All adult patients with unilateral limb cellulitis were eligible; the key exclusion criteria were antibiotic treatment for longer than 48 hours, previous *Clostridium difficile* infection, past MRSA carriage, allergy to either penicillin or clindamycin (self-reported or from their medical records) and pre-existing diarrhoea. Patients with obvious abscesses were not eligible. We collected data on randomised and non-randomised participants as specified by CONSORT (figure 1).

All participants were given flucloxacillin orally or intravenously, and the dose and route were decided by the clinical team treating the patient. The minimum dose and duration of flucloxacillin specified in the protocol was 500 mg four times per day orally for 5 days. Participants on other β-lactams prior to recruitment, for example, co-amoxiclav, were switched to flucloxacillin.

The dose of clindamycin in this trial was 300 mg four times per day orally, which has been used in a large trial of skin and soft tissue infections,^23^ and the duration was 2 days. The duration was designed to achieve adequate tissue levels of clindamycin while minimising the side effects. The protocol specified that clindamycin or placebo was to be started within 48 hours of starting the β-lactam. This period was a compromise between giving the patient adequate time to consider the trial and minimise the duration of any preceding β-lactam antibiotic, which might reduce any effect of clindamycin.

### Randomisation and blinding

Randomisation was web-based with blocks of eight and stratified by age (<65 and >64) and part of the week to allow for variation in follow-up caused by weekends. The randomisation system provided a study number and study drug bottle number. Study medication was prepared and dispensed by the University Hospitals Bristol Pharmacy which provided batches of the medication bottles to the study sites. Both clindamycin and placebo were formulated and supplied in identical capsules sealed in identical medication bottles. Only the pharmacy kept the code of whether the bottle contained clindamycin or placebo. The pharmacy had no access to the clinical data or patient identification unless unblinding was required. The study nurses, the statistician, and all investigators and participants were blind to whether the bottle contained clindamycin or placebo.

### Procedures

The study schedule consisted of three face-to-face visits, at baseline, days 5 and 10, with a telephone follow-up at day 30. At the first three visits, we measured standard observations (temperature, pulse, blood pressure and respiration rate). We also took blood for a full blood count, renal function, C reactive protein and albumin. We estimated the affected skin area of the limb using a scoring system similar to that used in psoriasis^24^ from which we calculated the percentage of body skin area. We measured the limb circumference at its greatest over the affected area and the highest temperature from the affected area and took comparable measurements from the unaffected limb, if available. The temperature measurements were made with an infrared thermometer,^25^ limb circumference with a disposable tape measure and pain scores were measured using a visual analogue scale. A record of adverse events was made at each visit. The actual number of days after randomisation on which follow-up occurred was variable. The day of follow-up was dependent on the patients' circumstances and, rather than lose a patient to follow-up, we asked patients to attend whenever they could, while still maintaining the target of 4 days post randomisation. We asked patients to return the study drug containers and counted the remaining capsules.

We collected additional information, including Euroqol (EQ-5D-5L) and Health Today scores, at baseline and day 30 for a health economics analysis. We asked patients at each visit and at the day 30 telephone follow-up whether or not they considered themselves to be back to their normal activities. At baseline, we recorded the time between the onset of systemic features, if reported, and local features. We recorded the time that antibiotics were first taken. We asked the patients about previous surgery, trauma or cellulitis of the affected limb and whether they had been diagnosed as having lymphoedema or diabetes mellitus as these are recognised risk factors.^26^

### Outcomes

The primary outcome was improvement at the day 5 follow-up visit. This was defined in the protocol as being afebrile (<37.5°C) and either having a reduction in limb swelling (measured by limb circumference) or a reduction in erythema (measured by skin surface temperature) of 0.2 SDs or more for both local measurements. The reduction in limb swelling and limb temperature was determined using the difference between affected and unaffected limbs to reduce confounding by ambient temperature, clothing and posture. These three clinical features were chosen because they could be measured accurately. Secondary outcomes included resolution of systemic features, resolution of inflammatory markers, recovery of renal function, reduction in the affected area, decrease in pain, return to work or normal activities and the absence of increased side effects.

### Statistics

The estimated number improving in the placebo group at first follow-up was 80%. A two-group continuity-corrected χ^2^ test with a 0.05 two-sided significance level will have an 80% power to detect the difference between 80% improving in the placebo group and 90% improving in the clindamycin group (OR of 2.250) when the sample size with complete data in each group is 219 (total 438). We were not able to make any estimates of the recruitment rate and likely losses to follow-up as there was no suitable source of data; the only UK trial of cellulitis in an emergency department (the primary source of patients for this study) was reported in 2005 and used parenteral antibiotics.^27^

Confirmation of the determination of the primary outcome was made by the Trial Steering Group during the trial. There was no interim analysis and there were no prespecified subgroup analyses. The decision to undertake subgroup analyses was made after the analysis of the primary outcome. The trial was stopped at 26 months, with 410 patients randomised, for funding reasons.

Data were analysed on an intention-to-treat (ITT) basis. Fisher’s exact test, with ORs, was used to determine whether there was a difference in those improving in each group at first follow-up (day 5). The analysis of the secondary outcomes adjusted for some baseline imbalances between the treatment allocations (namely baseline total affected area, difference between baseline affected and unaffected limb temperature, difference between baseline affected and unaffected limb circumferences and the logarithm of neutrophil count). The analysis of the primary outcome was also repeated, adjusting for these factors. Continuous outcomes were analysed using analysis of covariance (ANCOVA) and also adjusted for the baseline value of the outcome as well as the baseline imbalances. For dichotomous outcomes, logistic regression analysis was used to compare the adjusted odds of the outcome by treatment group. For continuous outcomes, there were some occasions where the distribution of the data was such that the logarithm of the outcome was used in the ANCOVA, and there were also a few occasions when the assumptions for the ANCOVA were not met, so it was necessary to use a Mann-Whitney test to compare the allocations. Medians have been reported instead of means where the data are markedly skewed.

## Patient involvement

Patients were not involved in designing the study apart from the patient information sheet. We used qualitative study data collected previously, which highlighted patient comfort as important, to inform the choice of outcomes.^28^ ^29^ The day 30 telephone consultation asked for feedback and comments on the conduct of the study. Towards the end of the study, we arranged a meeting and an online survey focusing on patients' symptoms and non-pharmacological interventions.

## Results

### Baseline

In total, 2444 patients with a diagnosis of cellulitis were screened for eligibility. Four hundred and ten patients were randomised, ranging from 18 to 95 years. Nine patients were subsequently found to be ineligible because they had received more than 48 hours of antibiotics (five patients) or the diagnosis was incorrect (four patients). The flow chart (figure 1) summarises the numbers of patients screened for eligibility and numbers present at each follow-up. All patients randomised are included in the ITT population. The baseline characteristics of the randomised patients are summarised in table 1; not every patient had a complete set of baseline data. The clindamycin allocation patients were slightly younger and had less severe cellulitis. Overall, 55 (13%) patients had a history of previous surgery, 63 (15%) of trauma to and 132 (32%) of cellulitis of the affected limb. Nineteen (5%) had a diagnosis of lymphoedema and 36 (9%) of diabetes mellitus.

**Table 1:** Baseline characteristics of the randomised patients

	Clindamycin (n=203)	Placebo (n=207)
Mean (SD) age in years	47.7 (18.4)	50.5 (16.9)
Male	129 (64)	149 (72)
Leg affected	150 (74)	149 (72)
Duration of local features before starting study drug (days); median (IQR)	2.1 (2.1)	2.0 (2.1)
Duration of preceding antibiotics before starting study drug (hours); median (IQR)	1.9 (19.0)	6.5 (23.5)
Temperature (°C); mean (SD)	36.8 (0.5)	36.9 (0.7)
Pulse (bpm); mean (SD)	81 (15)	81 (14)
Systolic blood pressure (mm Hg); mean (SD)	131 (20)	128 (18)
Affected skin area as percentage of body surface area; median and IQR	4 (4)	4 (6)
Difference in circumference between affected and unaffected limb (cm); mean (SD)	2.5 (2.1)	2.9 (2.1)
Difference in surface temperature between affected and unaffected limb (°C); mean (SD)	2.5 (1.9)	2.7 (1.6)
Neutrophil (×10^9^/L); median (IQR)	6.3 (4.6)	7.0 (4.9)
Lymphocyte (×10^9^/L); median (IQR)	1.6 (0.7)	1.5 (1.1)
Urea (mmol/L); median (IQR)	5.0 (2.1)	4.9 (2.1)
Albumin (g/L); median (IQR)	39 (7)	38 (8)
C reactive protein (mg/L); median (IQR)	23 (80)	54 (119)
Pain score (VAS); median (IQR)	5 (4)	5 (4)
SIRS score ≥1*	83/200 (42)	96/207 (46)

Figures are numbers of patients (percentage) unless otherwise stated.

Not every patient had every characteristic recorded.

One per cent of the total body skin area is ∼170 cm^2^; 10% of the total body skin area is approximately equal to the area of one arm or half the area of a leg.

*SIRS criteria: one point each for temperature <36°C or >38°C, pulse >90, respiratory rate >20, WCC<4 or >12×10^9^/L (WCC count derived from neutrophils plus lymphocytes plus 1).

SIRS, systemic inflammatory response syndrome; VAS, visual analogue scale; WCC, white cell count.

### Withdrawal from the study and non-attendance at day 5

Overall, 48 patients were actively withdrawn from the study, either at their own request or their physician's: 27 in the clindamycin allocation and 21 in the placebo allocation.

Six patients were found to be ineligible immediately after randomisation and did not receive the study drug (five clindamycin and one placebo). The most common reason for active withdrawal from the study was that the patient was given open-label clindamycin either unintentionally (4 patients) or intentionally (14 patients), 9 in each allocation. One of these patients was unblinded, the only one from the trial, at the request of the clinical team. Four patients had the diagnosis changed, three in the clindamycin allocation. Three patients were withdrawn because of an adverse event, one in the clindamycin allocation. Eleven patients withdrew their consent, eight in the clindamycin allocation. Seven patients withdrew for other reasons, three in the clindamycin allocation. Four patients given open-label clindamycin before day 5 are included in the analysis of primary outcome: two in the clindamycin allocation and two in the placebo allocation.

We looked at the baseline characteristics of those patients who did not attend the day 5 follow-up and compared them with those who did by univariate analysis. We found that the non-attendees, as a group, were significantly younger; the mean age of attendees was 50.8 and that of non-attendees was 41.4 years (p<0.001) Attendees were significantly more likely to have leg cellulitis than non-attendees: 76% vs 62% (p=0.02). Attendees were less likely to have a systemic inflammatory response syndrome (SIRS) score ≥1; 41% vs 59% of non-attendees (p=0.006). There were no differences in the baseline characteristics of the non-attendees between the study drug allocations.

### Primary outcome and day 5 follow-up

We were able to calculate the primary outcome for 328 patients. To be evaluable, patients had to have a body temperature measurement and a measurement of either limb circumference or limb surface temperature. Those with missing data on temperature at day 5, or the three with missing unaffected limb data, were categorised as having missing data for primary outcome. Patients with temperature data, but with missing data on both limb circumference and limb temperature, were categorised as not improved, rather than missing. Patients with temperature data, and with either a measure for change in circumference or change in limb temperature, were categorised according to the two out of three data items they had available. The median number of days to follow-up was 4.0 for clindamycin and 4.1 for placebo.

Within the evaluable population, there was no significant difference in improvement which was 87% for clindamycin and 81% for placebo; OR 1.55 (95% CI 0.81 to 3.01), p=0.17 (table 2). Adjusting for baseline differences did not alter the primary outcome result. As a proportion of those patients randomised improvement occurred in 67% of those on clindamycin compared with 68% of those on placebo; OR 0.97 (95% CI 0.63 to 1.50), p=0.92.

**Table 2:** Primary outcome

	**Clindamycin (n=203)**	**Placebo (n=207)**	
Insufficient day 5 data*	47	35	
Not improved	20	32	
Improved, as a proportion of those evaluable	136/156 (87)	140/172 (81)	OR 1.55 (95% CI 0.81 to 3.01) p=0.17
Improved, as a proportion of the randomised population	136/203 (67)	140/207 (68)	OR 0.97 (95% CI 0.63 to 1.50) p=0.92

Figures are numbers of patients (percentage).

*Either did not attend follow-up or were missing data.

### Route, dose and duration of flucloxacillin

The quality of the data on the duration and route of flucloxacillin during the trial was poor. We had not set out to collect data on dose because we assumed a standard dose of oral flucloxacillin, but we later discovered that some hospitals used higher doses. Consequently, we have not included this in any of the analyses presented here.

### Subgroup analyses

We did not specify any subgroup analysis in the study protocol but in order to clarify whether there might have been a positive effect from clindamycin in a subpopulation and a negative, or neutral effect, in another subpopulation, we undertook subgroup analyses. We looked at severity as defined as having a SIRS score of 0 or more than 0; duration of local features (area, skin temperature and swelling) of between 48 and 84 hours, or <48 hours prior to randomisation; duration of antibiotics prior to the study drug of >12 or <12 hours. No statistically significant difference in improvement in any subgroup was found.

### Compliance

The majority of the study drugs were taken; 148/159 (93%) in the clindamycin group and 159/176 (90%) of the placebo group had zero capsules remaining. The mean number of capsules remaining of the 11/159 in the clindamycin allocation was 6.5 and of the 17/176 in the placebo allocation was 4.4 out of 16 capsules.

### Day 10 follow-up and secondary outcomes

Fifty-four patients who attended at day 5 did not attend at day 10. The non-attendees were younger but otherwise similar to those who attended day 5. There was no significant difference in any secondary outcome measures except a slightly lower mean blood pressure (systolic, 3 mm Hg) in the clindamycin allocation at day 10 and lower median lymphocyte counts in the clindamycin allocation at both days 5 and 10 (0.18 and 0.19×10^9^/L, respectively). The median number of days to follow-up was 9 in both allocations. Table 3 summarises the secondary outcomes at days 5 and 10.

**Table 3:** Secondary outcomes

Time point	Clindamycin	Placebo	p Value
Temperature (°C); mean			
Baseline	36.8	36.9	
Day 5	36.6	36.5	0.07
Day 10	36.5	36.5	0.74
Pulse (bpm); mean			
Baseline	80	80	
Day 5	78	76	0.20
Day 10	78	77	0.76
Systolic blood pressure (mm Hg); mean			
Baseline	131	129	
Day 5	129	131	0.19
Day 10	128	131	0.02
Affected skin area as percentage of body surface area; median			
Baseline	4	5	
Day 5	2	2	0.28
Day 10	1	1	0.67
Difference in circumference between affected and unaffected limb (cm); mean			
Baseline	2.67	2.84	
Day 5	2.04	2.18	0.74
Day 10	1.42	1.77	0.90
Difference in surface temperature between affected and unaffected limb (°C); mean			
Baseline	2.49	2.67	
Day 5	1.15	1.57	0.24
Day 10	0.87	1.13	0.65
Neutrophil (×10^9^/L); median			
Baseline	5.97	6.95	
Day 5	4.24	4.43	0.95
Day 10	4.18	4.32	0.86
Lymphocyte (×10^9^/L); median			
Baseline	1.51	1.50	
Day 5	1.70	1.88	0.01
Day 10	1.75	1.94	0.01
Urea (mmol/L); median			
Baseline	5.0	4.9	
Day 5	4.8	5.0	0.09
Day 10	4.9	5.2	0.37
Albumin (g/L); median			
Baseline	38	38	
Day 5	38	37	0.82
Day 10	39	38	0.63
C reactive protein (mg/L); median			
Baseline	22	57	
Day 5	10	16	0.43
Day 10	5	6	0.20
Pain score (VAS); median			
Baseline	4.5	5	
Day 5	2	2	0.30
Day 10	0	1	0.61

Figures are values at each time point (means or medians).

The baseline values are of those patients who attended day 5. The baseline values for those attending day 10 are similar to the day 5 baseline values.

All p values are from ANCOVA (adjusted for baseline) except for pain VAS score at day 5 (Mann-Whitney U test).

ANCOVA, analysis of covariance; VAS, visual analogue scale.

### Back to normal activities

At day 5, 76/158 (48%) of the clindamycin allocation were back to normal activities compared with 74/175 (42%) of the placebo allocation (OR 1.09 (CI 0.66 to 1.78), p=0.74). At day 10, 77/133 (58%) of the clindamycin allocation were back to normal activities compared with 82/153 (54%) of the placebo allocation (OR 1.13 (CI 0.67 to 1.89), p=0.65). At the day 30 follow-up, the median duration from recruitment was 38.4 days in both allocations. Two hundred and fifty patients had a response to the question on their return to normal activities; 99/121 (82%) in the clindamycin allocation were back to normal activities compared with 104/129 (81%) in the placebo allocation (OR 0.90 (95% CI 0.44 to 1.84), p=0.77). The ORs are adjusted ORs adjusting for total affected area, difference in limb circumference, difference in limb temperature and neutrophil at baseline.

### Adverse events

Diarrhoea was the most common adverse event in the clindamycin allocation with 34/158 (22%) of the clindamycin allocation reporting it compared with 16/172 (9%) in the placebo allocation (OR 2.7 (95% CI 1.41 to 5.07), p=0.002; table 4). Adverse events resulted in three patients leaving the trial before day 5. Any hospital admission, for any reason, after recruitment and during the first 10 days of follow-up was recorded as a serious adverse event (SAE). There were 23 SAE (8 in the clindamycin allocation and 15 in the placebo allocation) and none were thought to be related to their study drug treatment. No case of *C. difficile* infection was reported. Two patients died before day 30 of causes unrelated to cellulitis or their treatment (one myocardial infarction and one pulmonary embolus), both of whom were in the placebo allocation.

**Table 4:** Adverse events or reactions

	Clindamycin	Placebo	p Value
*Reported at day 5*	*n=160*	*n=176*	
Rash	3 (1.9)	8 (4.6)	0.22
Diarrhoea	34 (21.5)	16 (9.3)	0.002
Any adverse event (including rash and diarrhoea)*	46 (28.9)	27 (15.6)	0.004
*Reported at day 10*	*n=135*	*n=151*	
Rash	2 (1.5)	10 (6.7)	0.04
Diarrhoea	17 (12.8)	8 (5.3)	0.04
Any adverse event (including rash and diarrhoea)*	19 (14.1)	19 (12.6)	0.73

A few patients on both follow-up days had missing data.

Figures are numbers of patients with an adverse reaction or event (percentage).

*Other events were: admission to hospital, nausea or vomiting, feeling light-headed or dizzy, lip swelling.

## Discussion

The results of this clinical trial do not provide evidence that any feature of cellulitis was improved by the addition of clindamycin. We collected a wide array of objective data in order to detect particular effects of clindamycin which might be related to toxin production and none showed any effect. The range of severity in the study patients was wide; we included those both on oral and intravenous flucloxacillin therapy. We looked to see whether clindamycin's effects may be only evident in those with more severe disease, in those who had a shorter duration of features or in those who had antibiotic treatment earlier. We could find no evidence that any of these circumstances made the addition of clindamycin beneficial. However, we did find that the proportion of patients in the clindamycin allocation with diarrhoea, up to day 5, was double that of the patients receiving placebo.

This study has some weaknesses; about 20% of patients did not attend the first follow-up but this is a feature of an acute infection which, in many people, heals rapidly, and of the population attending emergency departments. We were unable to find any significant differences in attendance between the two study allocations that could not be accounted for by the marginal differences in severity. We were unsure of what duration we should allow between initial antibiotic therapy and clindamycin but we could find no significant difference in outcome when we compared those having started antibiotics within 12 hours with those between 12 and 48 hours. We were also uncertain about the appropriate duration of clindamycin to detect an effect if it was present. We did detect an effect, an adverse one, diarrhoea, within the 48-hour duration.

This study was developed as a result of a south west England regional review of treatment of proven GAS infection which was undertaken in 2011. As part of this review, we could find no evidence of benefit of the addition of clindamycin despite its widespread and increasing use. This led us to look at a way of testing the hypothesis that clindamycin's protein-inhibiting activity is of benefit in GAS infection. There had been no previous studies on adjunctive clindamycin in cellulitis and we thought this study would be helpful in the management of cellulitis and invasive GAS infections. This study was designed to test the hypothesis that a toxin-inhibiting antibiotic, of which clindamycin is the exemplar, is effective in streptococcal infections. Cellulitis is a common streptococcal infection with easy-to-measure objective features and has been used in this trial as a representative streptococcal infection. We think it is reasonable to generalise the results of this trial to other streptococcal infections in the absence of specific trials.

The in vitro evidence that supported clindamycin's benefits is conflicting and the clinical evidence of benefit in invasive GAS infections has the biases intrinsic in these retrospective and prospective studies:^17^ ^18^ patient selection, patient treatment, recall and publication. The principal reason for the unsupported use of clindamycin in cellulitis is a widespread misunderstanding of the natural history of the condition. The local features of cellulitis will often progress (or at least not improve) for several days after presentation. An increasing affected area with mild fever and rising C reactive protein leads both the patient and physician to believe that the antibiotic treatment is failing; this is despite the patient's systemic symptoms of rigors, nausea and anorexia improving. The addition of clindamycin or change to intravenous therapy is consequently and spuriously attributed as being responsible for the patient's improvement. The fact that 14 patients were intentionally given clindamycin and withdrawn from the trial is a reflection of the belief in clindamycin's powers. It is probable that by the time the patient presents to a healthcare professional with local signs of cellulitis, the various toxins have bound to their targets and initiated the exaggerated inflammatory response which is part of the cause of skin damage. At this point, the *Streptococcus* is replicating and susceptible to β-lactam antibiotics, or no longer viable, so the addition of another antibiotic will have no beneficial effect. The mechanisms are similar to those of envenoming,^30^ and it is unsurprising that drugs thought to reduce toxin production given days later are unlikely to be effective.

The Cochrane review of interventions in cellulitis found no difference in outcome, however measured, between treatment with a β-lactam versus either macrolides or streptogramin.^10^ A large trial comparing dicloxacillin with clindamycin in the treatment of skin infections found no difference in efficacy.^23^ These studies support the outcome of this trial because if toxin inhibition was of importance, then the superiority of clindamycin should have been evident.

This clinical trial has shown that adjunctive clindamycin has no effect on improving outcome in cellulitis. Its only real effect is increasing the likelihood of diarrhoea with a rate comparable to that previously recorded.^23^ ^31^ It is also likely that there is no benefit in invasive GAS disease. Any further use of adjunctive clindamycin and other protein-inhibiting antibiotics, either concurrently or sequentially, for the treatment of streptococcal and staphylococcal infections should only be within a clinical trial.
